# An Image Segmentation Based on a Genetic Algorithm for Determining Soil Coverage by Crop Residues

**DOI:** 10.3390/s110606480

**Published:** 2011-06-17

**Authors:** Angela Ribeiro, Juan Ranz, Xavier P. Burgos-Artizzu, Gonzalo Pajares, Maria J. Sanchez del Arco, Luis Navarrete

**Affiliations:** 1 Centre for Automation and Robotics (CAR), CSIC-UPM, 28500 Arganda del Rey, Madrid, Spain; E-Mails: jranz@telefonica.net (J.R.); xpburgos@gmail.com (X.P.B.-A.); 2 Department of Software Engineering and Artificial Intelligence, Faculty of Computer Science, Complutense University, 28040 Madrid, Spain; E-Mail: pajares@fdi.ucm.es (G.P.); 3 Madrid Institute for Research and Rural Development, Agriculture and Food (IMIDRA), Finca El Encín, 28800 Alcalá de Henares, Madrid, Spain; E-Mails: maria.sanchez.arco@madrid.org (M.J.S.A.); luis.navarrete@madrid.org (L.N.)

**Keywords:** computer vision, conservation agriculture, estimation of coverage by crop residue, genetic algorithms, texture segmentation

## Abstract

Determination of the soil coverage by crop residues after ploughing is a fundamental element of Conservation Agriculture. This paper presents the application of genetic algorithms employed during the fine tuning of the segmentation process of a digital image with the aim of automatically quantifying the residue coverage. In other words, the objective is to achieve a segmentation that would permit the discrimination of the texture of the residue so that the output of the segmentation process is a binary image in which residue zones are isolated from the rest. The RGB images used come from a sample of images in which sections of terrain were photographed with a conventional camera positioned in zenith orientation atop a tripod. The images were taken outdoors under uncontrolled lighting conditions. Up to 92% similarity was achieved between the images obtained by the segmentation process proposed in this paper and the templates made by an elaborate manual tracing process. In addition to the proposed segmentation procedure and the fine tuning procedure that was developed, a global quantification of the soil coverage by residues for the sampled area was achieved that differed by only 0.85% from the quantification obtained using template images. Moreover, the proposed method does not depend on the type of residue present in the image. The study was conducted at the experimental farm “El Encín” in Alcalá de Henares (Madrid, Spain).

## 1. Introduction

All around the World, most farmers till the soil in order to prepare it for sowing. When tilling prior to sowing, the residues from the previous harvest (mulch and stubble), animal manure and weeds are buried at the same time that the soil is aerated and warmed up. However, this form of cleaning and stirring up the soil leaves it more exposed to erosion by wind and water. This makes farming a major cause of degradation of agricultural land and is a serious environmental problem worldwide. Conservation Agriculture [1] involves a set of agricultural practices and concepts organized around two basic principles:

1. Minimal disturbance of the soil and no-tillage, while leaving the soil covered with the residue from the previous harvest.
2. Conservation of permanent coverage of the soil, using own crops or cover crops, manure or mulch.

Conservation Agriculture proposes to apply minimum tillage, or eliminate it entirely, thereby contributing to the preservation of organic matter in soil and the reduction of erosion by wind and water. It follows that residue is, for Conservation Agriculture, a valuable resource in protecting the soil from the impact of erosion from precipitation and subsequent runoff. Retention of residue is therefore recommended as an important part of soil management. This does not imply the retention of excessive amounts of residue, but just the amount sufficient to protect the soil, the excess being useful as animal feed. Crop residues were initially classified along with the parameter that indicated the “dry weight per unit area of ground with cover” but it was soon shown that the percentage of soil covered by residue is better related to erosion control than the dry weight measurements [2]. As far back as 1993, some researchers [3] recommended the development and application of appropriate techniques for measuring the percentage of covered *versus* bare soil in order to improve the precision of research results in Conservation Agriculture, in addition to achieving adequate residue management and monitoring done by the farmer. It is therefore of great importance to be able to rely on mechanisms that can simply and accurately map the distribution of residue covering an area.

As with many tasks executed in the field, maps of zones (consisting of stubble, mulch, weed, *etc*.) are constructed from a systematic sampling in which information is gathered for some location/point on the ground in order to infer the remaining points by some interpolation method. In the case of crop residues, the experts cross the ground while executing a visual inspection of the zone. This evaluation is tedious and prone to estimation errors typical of tasks in which it is not possible (or is difficult) to review prior evaluations and in which perception has a tendency to adapt to the dominant state of affairs. In other words, an initial high estimate of residue coverage may have been considered to be average if the majority of data points sampled until the occurrence of that estimate, exhibited a high value for the residue.

The process of making estimates in the field can be improved if a sample of geo-referenced photographs is made so that a specialist can subsequently estimate in the lab the degree of crop residue coverage at each point. This approach to constructing a residue coverage/distribution map has a number of advantages:

1. Information gathering (sampling) can be done by an operator without having to take an entire team to the field.
2. The specialist can review the pictures and reexamine as often as necessary his evaluation criteria.
3. This opens the possibility of developing an application to automatically determine the amount of coverage in each photo, that is, at each sampling point.

Several techniques for mapping crop residue exist in the literature: visual estimate method, line-transect method, point intercept method, meter stick method, spiked wheel method and the photographic method. In fact, methods employed to date can be grouped in two categories: (1) traditional manual-visual methods [4–6] and (2) image analysis methods.

Among those methods that involve image analysis we encounter highly manual methods like the one proposed in [4], in which a slide is projected onto a screen with a grid overlaid on it followed by manual determination of the location of residue, or more recent methods which estimate the proportion of residue through comparison with residue templates for which this percentage is known. In this latter case, 10 to 20 images are used to establish the percentage of coverage by residue of a plot of field, with an error of about 25%. The work described in [7] includes images of residue patterns made by wheat, maize, sorghum and soybeans.

Another interesting technique is that proposed in [8]. In this case RGB images from a 30 frames per second video take are used. The authors then used an estimation method [9] based on the study of an image from a random selection of different pixels of that image whereby the classification algorithm uses artificially generated images simulating the residue of small and differently sized wooden slivers as templates. The video image, once digitized, is compared with the artificial template of wooden slivers to determine if the plots being examined contain residue. The initial estimations by the authors employed 0.2 mm wide by 0.2 mm high pixels and varying the pixel dimension yielded different values for the percentage of coverage. Clearly, the method is very dependent on how the simulation progresses when using wooden slivers.

In [10] the authors presented the development of a quantification method that focused on applying a threshold on the histogram derived from gray scale images, the objective being to achieve a binary image that isolated the residue. In a manner similar to the work described in [8], they randomly explored a series of pixels within an image such that the evaluation of larger number of pixels reduced the error. At an early stage, and based on the analysis of textures for differentiating bare and covered ground [9], they produced an algorithm that used specific operations on matrices in order to determine the texture of a continuous array of pixels. This algorithm never functioned adequately owing to the wide spread of existing textures which prevented its fast and precise execution. The same authors later generated artificial images in order to compare the performance of different methods for quantifying the coverage by residue [11].

On the other hand, in the last years remote sensing has supplied quite good results in the crop residue mapping [12–14], providing a rapid estimation of crop residue cover for big areas. But unlike traditional ground-based methodology, remote sensing is an expensive technique. In addition, aerial imagery and satellite data lack the necessary spatial resolution, and their acquisition depends heavily on weather conditions (e.g., clouds and fog). Even more, remote sensing derived residue products must be validated against ground reference measurements; it is therefore essential to have an approach that facilitates standardization and consistency in the ground data collection.

To sum up, the ground methods propounded so far are on the whole tedious and require, in the case of manual-visual methods, that a group of specialists travel to the field. The image analysis methods are in their turn also fairly manual. Within this context, we propose to address the quantification of crop residues with a two-stage strategy:

1. Photographic sampling in which geo-referenced images of the area are obtained with the aid of a GPS receiver. This stage does not require the presence of skilled personnel and can be executed by an operator, and it is also possible to obtain the images using equipment mounted on a vehicle. During the acquisition of images, it will not be necessary to control lighting.
2. Quantification in the lab of residue coverage in each image using an automatic segmentation process that isolates zones of residue in such fashion that the number of residue pixels *versus* the total number of pixels in the image can be counted (thereby yielding the percentage of coverage). The proposed procedure separates zones of residue from other elements of the image, such as shadows, soil, vegetation cover, *etc*. Once the residue coverage has been quantified for each image, it is possible to automatically generate by means of an interpolation procedure a map of the crop residue coverage for the entire sampled area.

This article presents the proposed segmentation process developed to automatically separate the zones within digital images covered by residue from the other elements present in the image. This procedure is the central component of a system for automatically generating maps of crop residue coverage (stage 2).

The next section details the proposed approach. Section 3 describes the tuning process carried out to optimize the segmentation parameters. Section 4 presents the most interesting results of this work while Section 5 presents the most relevant conclusions.

## 2. Experimental Section

### 2.1. Characteristics of the Set of Images Used

**Figure 1.**
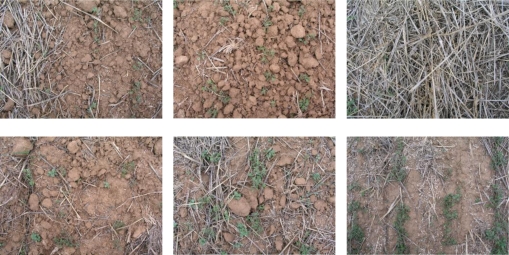
Examples of images taken at different sampling locations.

The study was conducted at the experimental farm “El Encín” of the *Instituto Madrileño de Investigación y Desarrollo Rural, Agrario y Alimentario* (IMIDRA, Alcalá de Henares, Madrid). The color images (RGB images) show zones with different levels of wheat residue (stubble) which were taken in the field under uncontrolled lighting conditions in February 2008, using a conventional Olympus C5050Z camera. Some examples are shown in Figure 1. Each image captures an area of 0.5 m by 0.5 m surrounding the sampling location/point. Fine tuning and verification of the procedure was done using 64 photographs that initially had a resolution of 2,560 × 1,920 pixels but were resampled to 640 × 480 pixels in order to reduce processing time. Figure 1 shows that in addition to residue, other elements such as vegetation cover, stones, soil, shadows, *etc*. have to be taken into account during the segmentation process.

### 2.2. Image Segmentation Procedure

In computer vision, segmentation is a process by which an image is partitioned into multiple regions (pixel clusters) [15]. The aim of segmentation is to obtain a new image in which it is easy to detect regions of interest, localize objects, or determine characteristic features such as edges. As a result, the image obtained by the segmentation process is a collection of disjoint regions covering the entire image whereby all the pixels of a particular region share some characteristic or property such as color, intensity, or texture. Furthermore, a standard digital camera captures the spectrum in three dimensions corresponding to the three primary colors: red (R) (in the range of wavelengths from 560 to 700 nm), green (G) (480 to 600 nm) and blue (B) (380 to 480 nm), which make up the final RGB image. Normally each pixel is coded using 24 bits, which implies 8 pixels per RGB plane, by which it is possible to code 256 intensities per pixel in each plane.

For the problem at hand the segmentation must be able to isolate the texture of the soil from the residue. The proposed procedure for the segmentation process uses a linear combination of the RGB planes of the original image (Equation (1)) followed by a subsequent thresholding (Equation (2))—a method that has delivered good results with similar problems [16–18], even though in the research described by these references, the objective was to segment weed zones. Specifically, the proposed segmentation process is divided into two steps:

1. Application of Equation (1) allows one to obtain, starting with the three matrices coding the RGB image, a gray scale matrix in which the value for the intensity of each pixel (*i,j*) will depend on the value of the intensity for that pixel in each plane, weighted by some constant coefficients (*c_r_*, *c_g_* and *c_b_*).
2. The binary image will be obtained by applying a threshold according to Equation (2) to the gray scale image obtained in the previous step.

(1) $$binaria(i,j)=\{\begin{array}{ll} 0 & \forall (i,j)|gris(i,j)\leq umbral\\ 1 & \forall (i,j)|gris(i,j)>umbral \end{array}$$

(2) $$binaria(i,j)\{\begin{array}{ll} 0 & \forall (i,j)|gris(i,j)\leq threshold\\ 1 & \forall (i,j)|gris(i,j)>threshold \end{array}$$

In short, the values of the coefficients *c_r_*, *c_g_* and *c_b_* as well the threshold value crucially determine the optimal segmentation—the best separation of residue from the remaining elements in the image. Fine tuning of these parameters has been achieved by means of a genetic algorithm—which will be explained in more detail in the next section—using 64 template images. The aim of the “genetic” tuning method is to determine the set of values (*c_r_*, *c_g_*, *c_b_*, *threshold*) by which the optimal segmentation can be achieved *i.e*., by which images most similar to the template images can be obtained.

When it comes to evaluating methods for the quantification of residue, we need a method for obtaining the real percentage of coverage by residue that a sample point of ground has. This is a control method for obtaining the reference value of a sample. One of the methods developed for obtaining the “true coverage” of residue, and one which is used sparingly because of it is extremely time-consuming, consists in projecting the image onto a screen on top of which a transparency is overlaid which can then be used to trace the zones of residue coverage. The transparency is then scanned and the number of black pixels is counted. The ratio of this number to the total number of pixels yields the residue coverage fraction with an error of +/− 1% [5].

**Figure 2.**
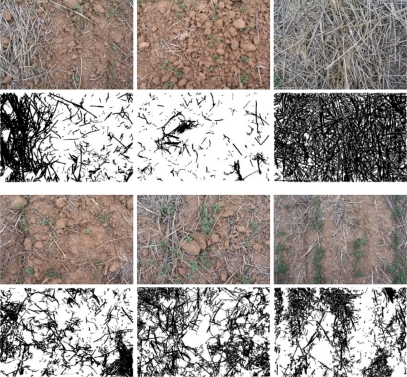
Images of sampled locations and binary template images manually produced by a specialist.

Applying some simplifications to the previous method yielded 64 starting templates that enable the fine tuning of the proposed segmentation procedure. To produce the templates, a specialist proceeded as follows: (1) Print the image at high resolution and quality (2) Lay a transparency on top of the image (3) Using a black marker, trace the residue observed on the image (4) Digitize the transparency at high resolution using a scanner and white paper as background. Figure 2 shows some examples of the templates that were produced.

### 2.3. Fine Tuning of the Segmentation Parameters

There is an extensive literature dealing with optimization algorithms, notable among these being the gradient methods which converge on the nearest solution following an increasing or decreasing gradient (maximization or minimization) without the ability to discern a local from a global solution. New heuristic methods have been proposed as alternatives during the last two decades, among which genetic algorithms stand out, having become very popular on account of their flexibility and ability to resolve complex and diverse problems. Genetic algorithms are part of the practice of Evolutionary Computation, which in addition to genetic algorithms includes evolutionary programming, evolutionary strategy, and genetic programming, in other words diverse methodologies for stochastic computation inspired by evolutionary biology.

Focusing on the genetic algorithms, these are defined as stochastic global optimization methods based on the principles of natural selection and evolution [19,20]. In accordance with Darwin’s theory of evolution, evolution in genetic algorithms proceeds by promoting the survival of the fittest. Basically, a population of “individuals”–in this case adequately coded potential solutions to problems—are made to evolve towards an optimal solution by means of the selective pressure exerted by selection operators or by crossover and mutation, using a cost or fitness function to measure the quality of solutions which iteratively proceed to the replacement of generations, in other words, the various operators and functions guide the search for a solution.

For the problem at hand the population of solutions is composed of individuals representing different combinations of values of the segmentation parameters (*c_r_*, *c_g_*, *c_b_*, *threshold*). The basic scheme behind genetic algorithms includes selection operators, crossover and mutation, and a binary representation of the parameters to optimize, although the method can be extended without major structural modifications, to any other alphabet in accordance with the nature of the problem. In our case, a non-binary encoding was elected so that the parameters *c_r_*, *c_g_*, *c_b_* are encoded using floating-point numbers and *threshold* is encoded using an integer value, thereby limiting size of a chromosome or individual. The initial population has been randomly generated.

Regarding the termination criterion, two termination conditions are established. Specifically the fine-tuning process will stop when: (1) a fitness value of the population reach a value under 0.05 or (2) the fitness does not improve after 50 generations.

The population is composed of 100 individuals. We used proportional selection, also known as *roulette wheel selection*, which is stochastic in nature. A two point crossover operator was selected as the operator to implement crossover. In other words, once two parents have been chosen, their children will grow initially as replicas of their parents and will only crossover with a probability fixed by the user. When recombination takes place the two point crossover process selects two points at random within the chromosome, based on which sequences of bits in each offspring are interchanged so as to produce new individuals. The crossover probability was fixed at 0.8. The elitism operator is then invoked to prevent the loss of good solutions. For the mutation operator we have chosen to use Gaussian mutation in which each gene (which in our case encodes the value of a single parameter) is added to a random value taken from a distribution having 0 median and a variance calculated as a function of the parameters *scale* and *shrink*. The parameter *scale* determines the deviation from the initial generation and *shrink* determines how the variance diminishes with each generation. In our case, the value of *scale* was fixed at 0.5 and *shrink* was also fixed at 0.5. Finally, the fitness function for each individual measures the degree of similarity between the binary image obtained from the segmentation process with the parameters coded by the solution individual (*A*) and the pattern image (*B*) (Equation (3)):

(3) $$S(A,B)=\frac{\sum_{i=1}^{n}\sum_{j=1}^{m}\left|A(i,j)-B(i,j)\right|}{nxm}$$

Equation (3) has values in the range [0,1] with 0 corresponding to complete similarity (identical images) and 1 corresponding to complete dissimilarity.

**Figure 3.**
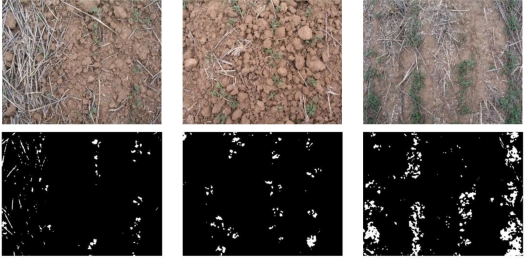
Original and binary images of weeds.

When segmentation parameters were being fine-tuned, some images were encountered that displayed areas covered by plant cover (see Figure 3) and this adversely affected the convergence of the fine tuning method. To resolve this, an extra step was added to the segmentation procedure with the aim of detecting and eliminating pixels associated with vegetation cover appearing in the image. Summarizing, the modified segmentation procedure proceeds as follows:

1. The vegetation cover in the initial image is isolated using Equations (1) and (2) together with the parameters proposed in [21], thereby obtaining a binary image. The results for several of the original images are shown in Figure 3.
2. Segmentation is applied to the initial image using Equations (1) and (2), with values for the constants *c_r_*, *c_g_*, *c_b_*, and *threshold* obtained from the tuning process, or with values coded in the solution individual when the fitness function is being evaluated.
3. The final binary image showing zones of residue coverage in white is obtained by subtracting the image generated in the first step from the binary image obtained in the second step.

Finally, in order to enhance the images obtained in the previous steps by reducing noise, we applied a 5 × 5 median filter which is the most effective method for reducing noise while preserving edges [15].

## 3. Results and Discussion

Two types of fine-tuning were carried out. In the first case, and to verify the suitability of the genetic algorithm, the fine-tuning of the segmentation parameters (*c_r_*, *c_g_*, *c_b_* *and threshold*) was done separately for each image. It was observed that the best segmentation parameters differed for each sample image. As a result, the best value for the fitness function - depending on the input image—varies between 0.08 and 0.24, which translates to a similarity between the segmented image and the template image varying between 76% and 92%.

In the second case the fine-tuning of the genetic algorithm used 20 images selected at random (the training set), the goal being to determine the segmentation parameters that yielded the best average performance for this set, as well as to subsequently verify the performance of the segmentation parameters when applied to the full set of 64 images. The values obtained as a result of fine tuning are shown in Table 1.

Applying these values to the set of 64 starting images, an average of 0.2138 was obtained for the fitness function. This corresponds to a similarity of 76.82% between the templates produced by the specialist and the images generated by the proposed segmentation method. Maximum similarity occurred at 92.13%.

It is important to notice that when the fine-tuning was carried out separately for each image, the initial population was randomly generated and the parameters *c_r_*, *c_g_*, *c_b_* could have taken on any real value. After studying the results of these initial experiments, we decided to incorporate restrictions for the values of the parameters based on their expected range of values. The aim was reducing the search space, improving the convergence of the fine tuning method. As a result, the parameters *c_r_*, *c_g_*, *c_b_* took on any real value in the range [−10, 10] while the *threshold* took on integer values in the range 0 to 255.

**Figure 4.**
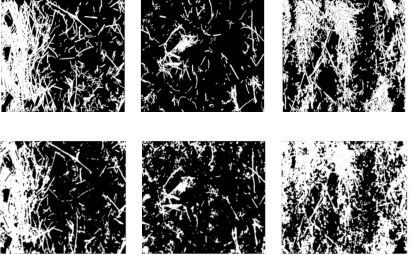
The first row shows negatives of the template images produced manually. The second row shows images obtained from the finely-tuned segmentation method.

Figure 4 shows various images produced by the segmentation process (second row) as compared to the template (first row). For the images shown the quantification errors are, from left to right, −4.42%, +5.61% and +9.35% respectively.

**Figure 5.**
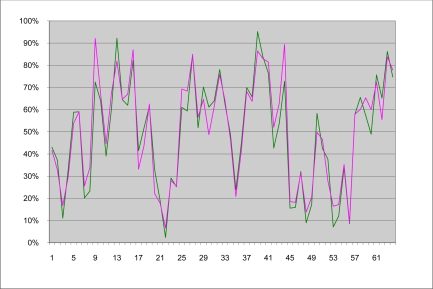
Plot of the percentages of coverage obtained from the template images (green) and from the computed images (pink).

**Figure 6.**
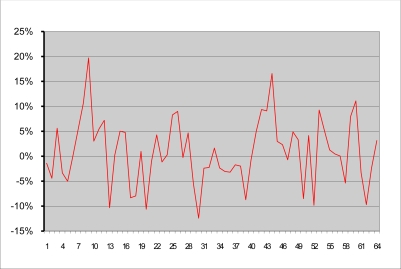
Plot of the differences in percentage of coverage between the template images and the computed images.

Figure 5 shows a comparative chart which plots for each of the 64 images (along the x-axis), their corresponding coverage (along the y-axis) obtained with the template and with the computed image produced by the finely tuned segmentation process. Figure 6 displays the percentage difference of the coverage, between the template images and the computed images. Most values lie within an error range of 5% and only few points exhibit errors exceeding 10%.

In many cases it is not necessary to have a map of the distribution of residue, as one can use the sample images to estimate the residue coverage. Using the 64 template images, a calculation of the coverage yields a total value of 49.63% for the sampled area. If the same calculation is done using the images obtained by applying the proposed segmentation process after it was finely tuned by the genetic algorithm, one achieves a coverage percentage of 50.48%. In other words, the automatic quantification method measures the coverage of residue with an error of 0.85% with respect to the manual approach. This difference in percentage of coverage is much less than the error sustained when doing pixel-to-pixel comparisons between the templates and computed images—the error in quantification decreases with the number of pixels. This is the effect of error compensation by which the quantification from some images err on the negative side and for some on the positive side. All in all, if one can count on a sufficient number of images, it is possible for the proposed method to produce a value for the quantification of coverage by residue that is very close to the real value.

Finally, regarding the number of generations, about 250 generations were necessary to reach one of the termination conditions for the independent fine-tuning of each image. Obviously, the situation was more complex when the objective was determining the segmentation parameters that yielded the best average performance for the 20 images of the training set. In this case, about 10,000 generations were necessary to reach a termination condition.

## 4. Conclusions

This paper has presented an automated method for determining the crop residue coverage of farmland following the harvest, by means of the segmentation of sample color images using genetic algorithms to fine tune the segmentation parameters.

The segmentation of residue appearing in images poses the problem of discriminating between this texture and other elements in addition to bare soil. For this reason the images that were taken without control of illumination display elements of a different nature such as vegetation cover, stones, shadows, *etc*.

The proposal to isolate zones of residue in an image consists of a 3-step segmentation process that include: the discrimination of zones of vegetation cover using a procedure developed previously by the research team; the generation of a binary image starting from the color RGB planes and a thresholding operation; finally the elimination in the final image of those pixels corresponding to zones of plant cover. The result is a binary image displaying only the zones of crop residue in white.

In order to fine tune the segmentation parameters, that is, the coefficients of the linear combination (of RGB values) and the threshold, a genetic algorithm was used to search for the best values. During fine tuning, 64 images were used and a template was manually produced for each one. These templates were used as a reference for establishing the fitness function value and for verifying the performance of the proposed method.

The results obtained were very good. The segmentation was achieved using images taken in conditions of uncontrolled lighting–that exhibited a worse case similarity with the template image of 76% and a best case similarity of 92%. Likewise, the value for the quantification of total coverage by residue of the sampled field was 49.63% using the template images and 50.48% using the computed images. In other words, one can determine percentage cover by crop residue of the sampled field with a difference of only 0.85% between the template images and the computed images.

Finally, the fine tuning method proposed is general so it can be easily used to adjust digital images of other crop residues. Furthermore, the wheat residue is a very common cover in Conservation Agriculture. Even more the residues of many other crops are very similar to wheat ones. In consequence, we can conclude that the presented work covers a wide variety of situations and crops, providing a general method.

## Figures and Tables

**Table 1.** Final values of the segmentation parameters for a training set of 20 images selected at random.

**Parameter**	**Value**
*c_r_*	−8.3675
*c_g_*	0.7128
*c_b_*	8.9926
*threshold*	93.316
